# A Case of Phthisis Apparently Cured

**Published:** 1896-02

**Authors:** Willam Pepper


### Selections and Abstracts.

---

### A CASE OF PHTHISIS APPARENTLY CURED.

By WILLAM PEPPER, M. D.

University Medical Magazine, 1895, vol. viii, p. 157.

The patient was a woman of 21 years, with a decided tubercular taint. In March, 1893, she suddenly began to lose flesh, had anorexia, deranged digestion, cough, and expectoration. The disease progressed so rapidly that when first seen, in the latter half of the month, she was already bedridden. She was found extremely emaciated. Constant irregular fever, with high evening rise. Night-sweats were profuse and exhausting. The slightest cause would provoke violent vomiting. The expectoration was thick, tenacious, heavy, and of an average amount of eight ounces. There were increased fremitus, dullness on percussion, and bronchial breathing, with fine, crackling rales over the right apex and the left base. The sputum contained myriads of tubercle bacilli.

Egg-albumen, agreeing better with her stomach than any other food, was taken daily to the amount of the albumen of two dozen eggs. Hypodermatically, every two hours, there was given $\frac{1}{100}$ grain of strychnine nitrate combined with $\frac{1}{1000}$ grain of atropine sulphate; by mouth, every two hours, $\frac{1}{50}$ grain of strychnine nitrate combined with $\frac{1}{12}$ grain of the double chloride of gold and sodium and ½ grain of a vegetable digestive. She was also given cod-liver oil inunctions, with massage and passive movements, daily. After a few days, the gold and sodium was increased to ⅛ grain every two hours. When strychnine intoxication showed itself the drug was reduced, but later again increased, so that she was just inside the border-line of the drug's toxic action.

During April she gained flesh, the fever became less, night-sweats were less profuse, cough allayed, and the expectoration, still rich in bacilli, was much reduced. During the latter part of May she regained her normal weight of 125 pounds. The fever and night-sweats disappeared. Digestion normal and appetite good. Cough and expectoration very slight. Tubercle bacilli gone. The signs of consolidation disappeared only slightly, harsh breathing remaining over the affected parts. She was sent to the mountains with instructions to continue the general treatment and to practice deep and forced breathing. In September she returned in perfect health, weighing 134 pounds. She remained well until August, 1895, when she had a slight attack of pneumonia in the left base. The sputum resembled prune juice and was crowded with pneumococci and tubercle bacilli. Crisis on the eight day, and a few days later she was sent to the mountains.

She returned in ten days with anorexia, fever, cough, and expectoration full of tubercle bacilli. Weight, 114 pounds. Consolidation with moist rales over the left base. The same treatment was instituted as during the first attack. On November 1, 1895 her weight was 124 pounds; fever gone, and cough and expectoration almost disappeared. Since the last week in October there were no tubercle bacilli found. The consolidation was greatly reduced, and a few days later the patient was again sent to the mountains.

Noteworthy points in this case are: The sudden onset, quite like general miliary tuberculosis; the large number of bacilli; the rapid recovery, all the more remarkable with a pronounced tubercular family history; the complete disappearance of consolidation and bacilli; the acute reappearance after two years of the whole train of symptoms, with signs in the base of the left lung directly following an attack of pneumonia located in that vulnerable part; the large number of bacilli and their early complete disappearance in the second attack; the abatement of the consolidating process, and rapid recovery of the general health after the second attack; the absence from the treatment of all cough-medication and antiseptics, and the large doses of strychnine nitrate and the double chloride of gold and sodium, with which the system was kept literally saturated.—*American Medico-Surgical Bulletin.*

